# Variations in Physician Telemedicine Provision

**DOI:** 10.1001/jamanetworkopen.2023.21955

**Published:** 2023-07-06

**Authors:** Nate C. Apathy, Ram A. Dixit, Christian L. Boxley, Katharine T. Adams, Ethan Booker, Raj M. Ratwani

**Affiliations:** 1 National Center for Human Factors in Healthcare, MedStar Health Research Institute, Washington, District of Columbia; 2 Center for Biomedical Informatics and Data Science, MedStar Health Research Institute, Washington, District of Columbia; 3 Telehealth Innovation Center, MedStar Health, Washington, District of Columbia

## Abstract

This cross-sectional study assesses variation in the provision of telemedicine services among primary care physicians and quantifies the extent to which this variation may be explained by the individual physician vs temporal, patient, or visit factors.

## Introduction

Increased use of telemedicine during the COVID-19 pandemic has been well documented, including recent evidence of lower but steady use.^1^ Given that telemedicine is likely to remain a staple of the US health care system, several studies^1,2,3^ have sought to profile the patient populations that use telemedicine most consistently. However, observed telemedicine use is a function of both patient demand and physician supply. In surveys, physicians have been found to vary in their prevalence and intentions to continue providing telemedicine services.^4^ As a result, physician preferences alter patient access to telemedicine, but variation across individual physicians has not been well documented, nor has the role these preferences play in patient access. We aimed to quantify physician variation in telemedicine provision and the extent to which telemedicine use is explainable by the individual physician, adjusting for temporal, patient, and visit factors known to be associated with demand.

## Methods

This cross-sectional study analyzed adult primary care visits across MedStar Health, Stanford Health Care, and Intermountain Healthcare systems. Primary care visits were defined in the electronic health record and scheduling systems as completed outpatient visits with a primary care physician (internal, family, or geriatric medicine) who conducted at least 1000 visits between March 13, 2020, and December 31, 2021. Each visit was assigned a modality of in person or telemedicine, which included both telephone and video visits. This study was approved by the Georgetown–MedStar Health Institutional Review Board and deemed exempt from informed consent due to the use of deidentified patient and physician data. The study followed the Strengthening the Reporting of Observational Studies in Epidemiology (STROBE) reporting guideline.

To analyze physician variation in telemedicine provision and identify outlier physicians, we visualized weekly distribution in physician-level share of telemedicine visits. To quantify the extent to which physician preferences were associated with telemedicine visit modality, we constructed multilevel random-effects models at the visit level using a binary outcome (telemedicine vs nontelemedicine). We calculated intraclass correlation coefficients (ratio of between-group variance to total variance) to measure the proportion of variation in our outcome explained by each of the following variables: individual physician, week of visit, number of diagnoses the patient had at time of visit, evaluation and management level, whether the patient was new to the physician, insurance provider, and patient sex, age, and race and ethnicity. All analyses were conducted using R software, version 4.2.1 (R Foundation for Statistical Computing), and the lme4 package for multilevel modeling.

## Results

The sample consisted of 2 410 471 visits (617 765 [25.6%] via telemedicine) among 760 895 patients (58.9% female; 35.9% aged 51-70 years; 66.5% White) seen by 729 physicians (Table). We found substantial variation in telemedicine provision across physicians (Figure). While telemedicine provision declined over time, 237 physicians (32.5%) had at least 1 high-outlier week of telemedicine provision. In our multilevel models, the visit physician explained 7.7% of variation in telemedicine use compared with 7.8% explained by variation over time (ie, pandemic wave) and 7.6% explained by evaluation and management visit level (Table). Patient age, sex, race and ethnicity, insurance type, number of diagnoses, and new patient status cumulatively accounted for only 2.3% of the variation in telemedicine use; site explained 16.3% of the variation.

**Table.** Visit Sample Characteristics and Results From Multilevel Models

Characteristic	Individuals or visits, No. (%)				Variation in telemedicine use explained, %^a^
	Overall	Intermountain Healthcare	MedStar Health	Stanford Health Care	
Visits, No.	2 410 471	1 098 451	790 782	521 238	NA
Physicians, No.	729	284	274	171	7.7
Telemedicine visits, mean %	25.6	13.1	26.6	50.6	NA
New patients	90 667 (3.8)	27 533 (2.5)	31 685 (4.0)	31 449 (6.0)	0.7
Patient demographic characteristics					
Age group, y					
18-30	261 095 (10.8)	132 450 (12.1)	82 149 (10.4)	46 496 (8.9)	1.1
31-50	613 271 (25.5)	278 796 (25.4)	196 056 (24.8)	138 419 (26.6)	
51-70	865 396 (35.9)	363 417 (33.1)	317 049 (40.1)	184 930 (35.5)	
≥71	670 709 (27.8)	323 788 (29.5)	195 528 (24.7)	151 393 (29.0)	
Sex					
Male	991 077 (41.1)	482 659 (43.9)	294 197 (37.2)	214 221 (41.1)	0.2
Female	1 419 394 (58.9)	615 792 (56.1)	496 585 (62.8)	307 017 (58.9)	
Race and ethnicity					
Asian	164 116 (6.8)	19 916 (1.8)	24 476 (3.1)	119 724 (23.0)	0.1
Black	364 847 (15.1)	8133 (0.7)	329 362 (41.7)	27 352 (5.2)	
Hispanic	171 435 (7.1)	80 463 (7.3)	30 036 (3.8)	60 936 (11.7)	
White	1 603 891 (66.5)	957 384 (87.2)	372 563 (47.1)	273 944 (52.6)	
Other	106 182 (4.4)	32 555 (3.0)	34 345 (4.3)	39 282 (7.5)	
Insurance type					
Commercial	1 273 304 (52.8)	561 302 (51.1)	416 093 (52.6)	295 909 (56.8)	0
Medicaid	175 243 (7.3)	59 564 (5.4)	105 985 (13.4)	9694 (1.9)	
Medicare	899 644 (37.3)	449 047 (40.9)	262 146 (33.2)	188 451 (36.2)	
Other	62 280 (2.6)	28 538 (2.6)	6558 (0.8)	27 184 (5.2)	
Evaluation and management visit level					
1	15 962 (0.7)	611 (0.1)	15 214 (1.9)	137 (<0.1)	7.6
2	43 593 (1.8)	15 763 (1.4)	15 822 (2.0)	12 008 (2.3)	
3	654 240 (27.1)	318 765 (29.0)	159 879 (20.2)	175 596 (33.7)	
4	964 975 (40.0)	454 619 (41.4)	330 764 (41.8)	179 592 (34.5)	
5	67 455 (2.8)	16 436 (1.5)	24 424 (3.1)	26 595 (5.1)	
Non–evaluation and management visit	664 246 (27.6)	292 257 (26.6)	244 679 (30.9)	127 310 (24.4)	
Site^b^	NA	NA	NA	NA	16.3
Visit week^b^	NA	NA	NA	NA	7.8
Visit diagnoses^b^	NA	NA	NA	NA	0.2
Residual (unexplained) variation	NA	NA	NA	NA	58.3

Abbreviation: NA, not applicable.

^a^ Values in this column represent estimates of intraclass correlation coefficients from multilevel models with random effects for all covariates. Each coefficient was measured for 1 variable (ie, patient age group as a whole rather than patients aged 18-30 years specifically) and represents the amount of variation in the visit-level outcome of telemedicine use explained by each variable.

^b^ These variables were included in the multilevel models and thus had an estimated intraclass correlation coefficient that was already represented (eg, site) or had too many levels (eg, visit diagnoses) to succinctly list in the table.

**Figure.**
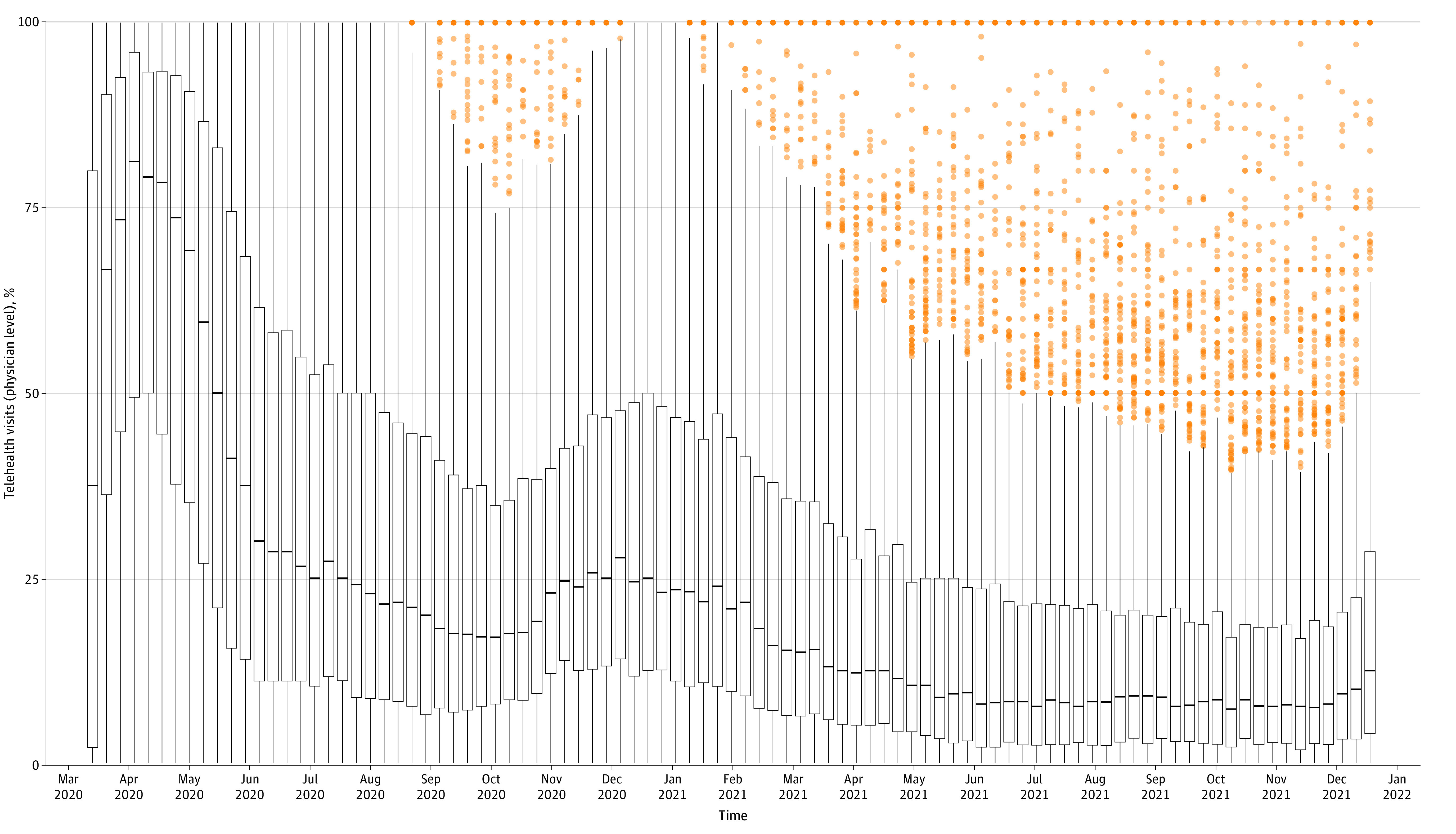
Variation Over Time in the Share of Visits Provided via Telemedicine Box plots show medians (represented by horizontal lines) and IQRs (represented by lower and upper boundaries of boxes indicating 25th and 75th percentiles, respectively). Orange dots represent outliers, defined as points that were outside of 1.5 times the within-week IQRs.

## Discussion

This cross-sectional study found that 32.5% of physicians in the sample continued to provide relatively high rates of telemedicine amid a general decline in telemedicine use. Previous assessments^2,4,5,6^ of physician perspectives on telemedicine have found that most intend to continue offering virtual services. However, findings from both our study and a recent survey study^3^ suggest waning physician use of telemedicine, largely due to the lack of physical examinations. Although our findings may not generalize to other health care systems or specialties outside of primary care, our study highlights a nontrivial share of physicians who do not reflect this decreasing pattern and instead exhibit persistently high rates of telemedicine use, suggesting generous telemedicine provision may be a differentiator for these physicians.

## Notes

Supplement.
Data Sharing Statement
