# Supplementary material for: Variations in Physician Telemedicine Provision
Source: JAMA Netw Open. 2023 Jul 6;6(7):e2321955. doi: 10.1001/jamanetworkopen.2023.21955 (PMC10326643; doi:10.1001/jamanetworkopen.2023.21955)
Supplement: Supplement. — Data Sharing Statement [file jamanetwopen-e2321955-s001.pdf]

Data Sharing Statement

Apathy. Variations in Physician Telemedicine Provision. *JAMA Netw Open*. Published July 06, 2023. doi:10.1001/jamanetworkopen.2023.21955

Data
Data available: No
